# Relative platelet reductions provide better pathophysiologic signatures of coagulopathies in sepsis

**DOI:** 10.1038/s41598-021-93635-5

**Published:** 2021-07-07

**Authors:** Daisuke Kasugai, Masayuki Ozaki, Kazuki Nishida, Yukari Goto, Kunihiko Takahashi, Shigeyuki Matsui, Naoyuki Matsuda

**Affiliations:** 1 Department of Emergency and Critical Care Medicine, Nagoya University Graduate School of Medicine, Tsurumai-cho 64, Showa-ku, Nagoya, Aichi Japan; 2 Department of Biostatistics, Nagoya University Graduate School of Medicine, Nagoya, Japan; 3 Department of Biostatistics, M&D Data Science Center, Tokyo Medical and Dental University, Tokyo, Japan

**Keywords:** Coagulation system, Risk factors, Outcomes research, Infectious diseases

## Abstract

In sepsis-associated coagulopathies and disseminated intravascular coagulation, relative platelet reductions may reflect coagulopathy severity. However, limited evidence supports their clinical significance and most sepsis-associated coagulopathy criteria focus on the absolute platelet counts. To estimate the impact of relative platelet reductions and absolute platelet counts on sepsis outcomes. A multicenter retrospective observational study was performed using the eICU Collaborative Research Database, comprising 335 intensive care units (ICUs) in the United States. Patients with sepsis and an ICU stay > 2 days were included. Estimated effects of relative platelet reductions and absolute platelet counts on mortality and coagulopathy-related complications were evaluated. Overall, 26,176 patients were included. Multivariate mixed-effect logistic regression analysis revealed marked in-hospital mortality risk with larger platelet reductions between days one and two, independent from the resultant absolute platelet counts. The adjusted odds ratio (OR) [95% confidence intervals (CI)] for in-hospital mortality was 1.28[1.23–1.32], 1.86[1.75–1.97], 2.99[2.66–3.36], and 6.05[4.40–8.31] for 20–40%, 40–60%, 60–80%, and > 80% reductions, respectively, when compared with a < 20% decrease in platelets (*P* < 0.001 for each). In the multivariate logistic regression analysis, platelet reductions ≥ 11% and platelet counts ≤ 100,000/μL on day 2 were associated with high coagulopathy-related complications (OR [95%CI], 2.03 and 1.18; *P* < 0.001 and *P* < 0.001), while only platelet reduction was associated with thromboembolic complications (OR [95%CI], 1.43 [1.03–1.98], *P* < 0.001). The magnitude of platelet reductions represent mortality risk and provides a better signature of coagulopathies in sepsis; therefore, it is a plausible criterion for sepsis-associated coagulopathies.

## Introduction

Sepsis, a major concern in the field of critical care, occurs from an uncontrolled and dysregulated immune response to infections, which causes multiple organ dysfunctions^1^. For decades, the survival rate of patients with sepsis has not shown a significant improvement, despite several randomized control trials (RCTs) on new treatment modalities and improved compliance with treatment guidelines^2–4^.

Platelets play an important role in the pathogenesis of sepsis and sepsis-associated mortality^5,6^. A decreased platelet count is observed commonly in sepsis and is associated with mortality^5,6^. During sepsis, thrombocytopenia is thought to occur due to an increased rate in platelet consumption via multiple pathways (e.g., the activation of platelet membrane receptors such as the toll-like receptor 4 and the protease-activated receptors^7,8^, hemophagocytosis^9^, and disseminated intravascular coagulation (DIC)^10^) rather than decreased platelet production^11^. From this perspective, the degree of platelet consumption reflects the pathophysiology and severity of the underlying coagulopathy.

Most international criteria for coagulopathy in sepsis do not consider the relative platelet reductions but focus on the absolute platelet counts^12–15^. However, given that the normal platelet count range is broad, similar platelet counts may not imply a similar extent of hemostatic derangement. There has been limited evidence to support the clinical significance of reductions in platelet counts in sepsis^13,16^. Limited sample sizes and inadequate adjustments for confounders in previous studies have made it unclear whether absolute counts and relative reductions are independently important. In this context, further research is necessary to understand the influence of relative platelet reductions and the absolute platelet counts on sepsis outcomes. Therefore, this study aimed to estimate the impact of relative platelet reductions and the absolute platelet counts on sepsis outcomes.

## Methods

### Data source and study population

We performed a retrospective observational study using the eICU Collaborative Research Database that comprised stratified random samples of patients (admitted between 2014 and 2015) from data repositories of 335 intensive care units (ICUs) from 208 hospitals in the United States, amounting to 200,859 ICU admissions in total. The details of the database are described elsewhere^17^. The eligible subjects were patients with sepsis at the time of ICU admission. The definition of sepsis was in accordance with the third sepsis definition^1^. Specifically, patients with documented or suspected infectious diseases, along with the evidence of organ dysfunctions (total Sequential Organ Failure Assessment [SOFA] score ≥ 2 points), were screened^1,18^. Those who lacked data on platelet counts at the time of ICU admission, were diagnosed with heparin induced thrombocytopenia (ICD-10 code: D75.82), died, or were discharged from the hospital within two days after their admission to the ICU were excluded from the primary analysis.

We used the REporting of Studies Conducted using Observational Routinely Collected Health Data (RECORD)^19^ statements for reporting this study.

### Ethics approval and consent to participate

Data used in this study were de-identified and released under the Health Insurance Portability and Accountability Act (HIPAA) safe harbor provision. The re-identification risk was certified as meeting safe harbor standards by Privacert (Cambridge, MA) (HIPAA Certification no. 1031219-2). Therefore, the ethical approval statement by the local Institutional Review Board (Nagoya University Hospital Institutional Review Board) and the requirement for informed consent were waived for this study.

### Relative platelet reductions and outcomes

Relative platelet reductions were calculated from the initial platelet counts on day 1 (within 24 h of ICU admission) to the minimum value on day 2 (24–48 h of ICU admission). Assessment of the additional time-interval was added for the secondary analysis using the platelet counts between days 2–3, days 3–4, days 4–5, days 5–6, and days 6–7, respectively. To avoid immortal-time bias, we excluded patients who died or were discharged from the hospital before the duration of interest.

The primary outcome was in-hospital mortality. The secondary outcome was the presence of coagulopathy-related complications, defined as complications of new hemorrhagic events and thrombotic events after day 2 of ICU admission (Table S1 in additional file 1). If an active diagnosis of hemorrhagic and thrombotic events was recorded before and after day 2 in these patients, the patients were not considered to be experiencing new complications.

### Covariates

The covariates used for the primary model included patient-level variables such as age, sex, body mass indexes, ethnicities, comorbid conditions obtained using the Charlson comorbidity index, focus of infections, nosocomial onset of sepsis, severity scores defined using Acute Physiology and Chronic Health Evaluation (APACHE) IV, number of ICU visits, and types of ICUs^20–22^ and hospital-level variables such as bed capacities, region of hospital locations, and teaching statuses of the hospital^23–26^.

### Statistical analysis

The continuous variables were expressed as medians with interquartile ranges (IQR) or means and standard deviations, as appropriate. The categorical data were analyzed using the χ^2^ test to calculate the unadjusted odds ratios.

For the primary analysis, a multivariate mixed-effect logistic regression analysis was performed to estimate the mortality odds of each range of relative platelet reductions (< 20%, 20–40%, 40–60%, 60–80%, and > 80%) and mortality odds considering the absolute platelet counts on day 1 (the categorization was based on the SOFA scores and treated as continuous variables). The hospital of admission was used as a random intercept to account for the clustering by hospital location^24,25^. Subgroup analyses were also performed for those who did and did not experience thrombocytopenia, shock, acute respiratory failure, and who did not experience coagulopathy-related complications. The interaction between the relative platelet reductions and each subgroup were also evaluated. For the further evaluation of the estimated effect of the relative platelet reductions and absolute platelet counts of different time intervals, spline regression analyses were conducted using a generalized additive model.

To further examine the association between the platelet count trajectories and coagulopathy-related complications, a multivariate logistic regression analysis was performed to estimate the odds of the relative platelet reductions and absolute platelet counts on day 2 for coagulopathy-related complications. Since the incidence of coagulopathy-related complications was rare, we did not adjust the hospital-level variables in this model. The optimal cutoffs of each variable were determined using the best Youden index for receiver operating characteristic (ROC) curve analysis^27^. Model discrimination for the in-hospital mortality and coagulopathy-related complications were assessed with area under the ROC curve using absolute platelet counts alone and added with the relative platelet reductions.

For handling missing data, we assumed that the missing data were conditional, based on the observed covariates (missing at random): multiple imputations were performed with multivariate imputations using the chained equations package^28^. For continuous, non-normal variables with upper and lower boundaries, we used predictive mean matching. The results of 10 imputed datasets were combined by averaging, and standard errors were adjusted to reflect both within- and between-imputation variability.

For the sensitivity analyses, we repeated our analyses: (1) with the complete case set, (2) using models with additional covariates for adjustment, and (3) under alternative criteria of patient selection, to test the robustness of the primary analysis. The alternative model included the following additional covariates, which had the potential to be related causally to the outcome and/or platelet reductions: initial type of antibiotics, heparin administration, and renal replacement therapy. For the alternative cohort, we selected patients with an explicit diagnosis of sepsis^29^. All the statistical analyses were performed using R version 4.0.0.

## Results

### Characteristics of the patients

**Figure 1:**
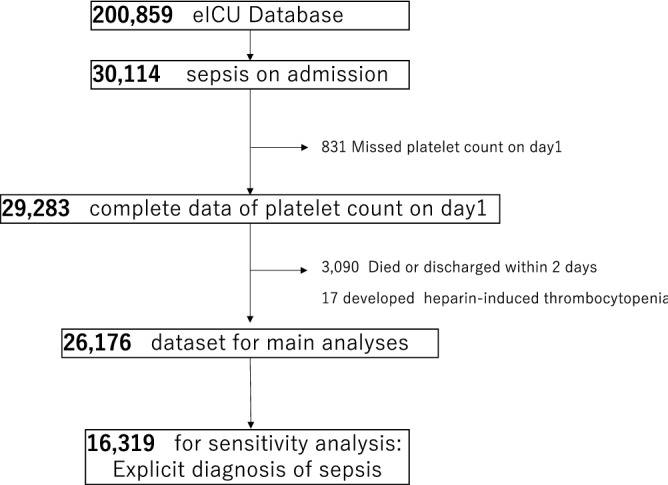
Flow diagram of patient selection.

**Figure 2:**
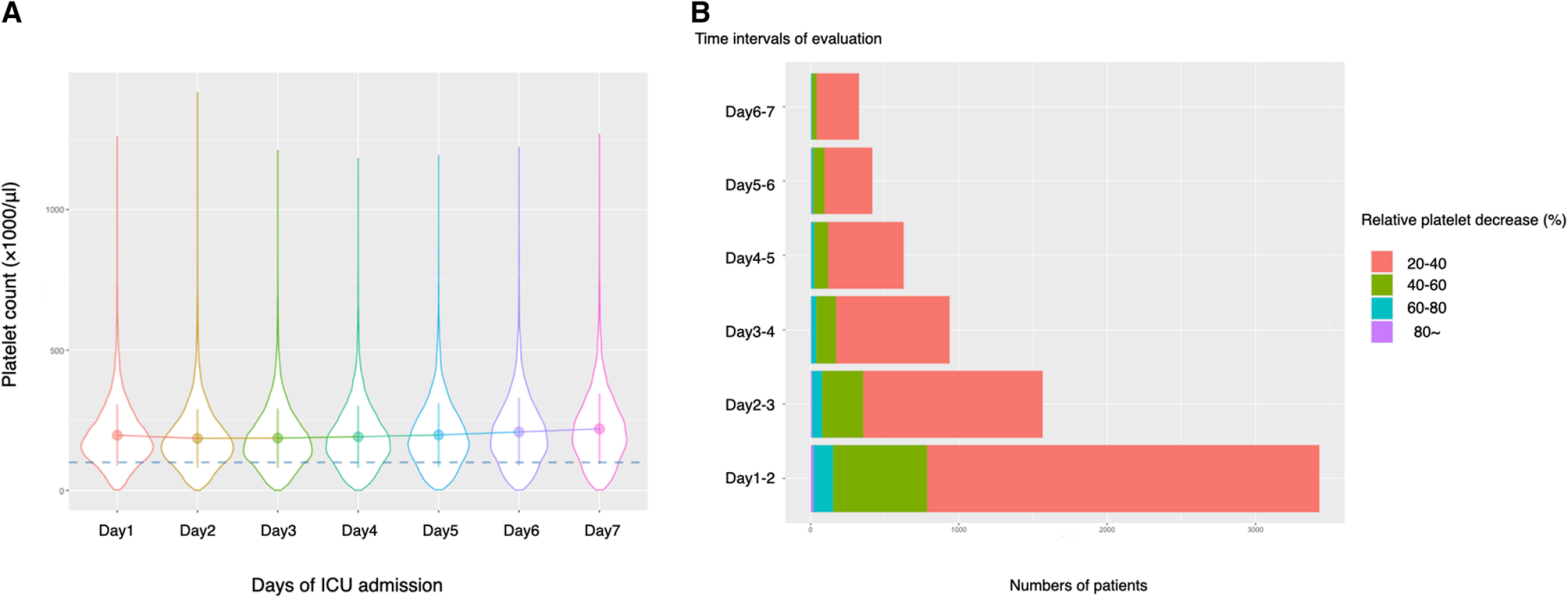
The trends and distribution of the platelet counts and the relative platelet reductions after the first seven days of ICU admission. (**A**) Trends and distribution of the platelet count. (**B**) Relative platelet reduction after the first seven days of ICU admission.

Among the 30,114 patients with sepsis, 26,176 (87.0%) were included in the primary analyses. The flow diagram of the patient selection for each dataset is shown in Fig. 1. A total of 3,938 were excluded: 831 (2.8%) due to the lack of absolute platelet counts on day 1 and 3,090 (10.3%) for death or discharge within two days, and 17(0.05%) for positive diagnoses of heparin-induced thrombocytopenia. The patient characteristics are summarized in Table 1 and are shown in detail (Additional file 2: Table S2). Patients aged > 60 years on average, were predominantly Caucasian (77.8%) and had pulmonary infections (52%). The median length of hospital stay was 7.4 days (interquartile range, 4.7–12.1 days), while the in-hospital mortality was 12.3%. New onset of coagulopathic complications after day 2 of ICU admissions was relatively rare (thrombotic complications: 139 [0.5%]; hemorrhagic complications: 145 [0.6%]). The distribution of the platelet counts in the first week of the ICU admission and incidence of the platelet reductions are shown in Fig. 2. A greater amount of platelet reductions was more likely to be experienced in the earlier phase.

**Table 1:** Baseline characteristics.

Total	26,176
Male sex, n (%)	13 514 (51.6)
Age, years, mean (SD)	66.16 (15.4)
Admission weight, kg, mean (SD)	84.07 (29.5)
**Ethnicity, n (%)**	
Caucasian	20 370 (77.8)
African American	2752 (10.5)
Asian	418 (1.6)
Hispanic	1057 (4.1)
Native American	216 (0.8)
Other/unknown	1363 (5.2)
**Focus of infection, n (%)**	
Abdominal	3596 (13.7)
Pulmonary	13,611 (50.9)
Soft tissue	1751 (6.6)
Urinary tract	3907 (14.6)
Others/unknown	3291 (12.3)
Charlson comorbidity index, median (IQR)	4 (2–6)
SOFA score on admission, median (IQR)	7 (5–10)
APACHE IV score, median (IQR)	65 (15–82)
Platelet count (day 1), × 10^3^/μL, median (IQR)	181 (125–251)
Platelet count (day 2), × 10^3^/μL, median (IQR)	174 (117–242)
**Treatment on day 1, n (%)**	
Mechanical ventilation	1925 (7.3)
Renal replacement therapy	1108 (4.2)
**New coagulopathy-related complications after day 2 of ICU admission, n (%)**	
Hemorrhage	145 (0.6)
Thrombosis	139 (0.5)
In-hospital death, n (%)	3208 (12.3)
Length of hospital stay, days, median (IQR)	7.4 (4.7–12.1)

*SD* standard deviation; *IQR* interquartile range; *SOFA* sequential organ failure assessment; *APACHE* acute physiology and chronic health evaluation.

### Relative platelet reductions, absolute platelet counts, and mortality

In the multivariate mixed-effect logistic regression analysis, the adjusted odds ratio (OR) for in-hospital mortality was 1.28 (95% confidence interval [CI], 1.23–1.32), 1.86 (95% CI, 1.75–1.97), 2.99 (95% CI, 2.66–3.36), and 6.05 (95% CI, 4.40–8.31) for 20–40%, 40–60%, 60–80%, and > 80% reductions, respectively, when compared with a < 20% decrease in platelets (Table 2). The estimated effect of the absolute platelet counts on day 2 was relatively small (OR, 1.2; 95% CI, 1.18–1.21, with an increase in the hematology component of the SOFA score). A synergetic interaction for mortality odds between relative platelet reduction and the resultant absolute platelet count was found (P = 0.006 for interaction). The presence of shock a correlated negatively with the mortality odds of the relative platelet reductions (*P* < 0.001 for the interaction). The presence of respiratory failure and development of coagulopathy-related complications did not modify mortality odds of relative platelet reduction (P = 0.737 and P = 0.339 for the interaction, respectively).

**Table 2:** Association of relative platelet reductions and platelet counts with in-hospital mortality for patients with sepsis.

Cohort	Crude Odds ratio (95% CI)	Adjusted odds ratio (95% CI)^a^	*P* Value
**Primary cohort**			
Relative platelet reduction (%)			
< 20	Reference	Reference	
20–40	1.69 (1.64–1.74)	1.28 (1.23–1.32)	< 0.001
40–60	2.98 (2.83–3.15)	1.86 (1.75–1.97)	< 0.001
60–80	5.47 (4.94–6.05)	2.99 (2.66–3.36)	< 0.001
≥ 80	13.56 (10.29–17.98)	6.05 (4.40–8.31)	< 0.001
Absolute platelet count on day 2^b^	1.29 (1.27–1.30)	1.20 (1.18–1.21)	< 0.001
Interaction with relative platelet reduction			
× thrombocytopenia^c^		1.08 (1.02–1.15)	0.006
× shock^d^		0.81 (0.77–0.86)	< 0.001
× respiratory failure^e^		0.99 (0.94–1.05)	0.737
× coagulopathy related complications		0.9 (0.75–1.1)	0.339
**Patient without resultant thrombocytopenia**^**c**^			
Relative platelet reduction (%)			
< 20	Reference	Reference	
20–40	1.70 (1.64– 1.76)	1.30 (1.24–1.36)	< 0.001
40–60	2.48 (2.32– 2.65)	1.64 (1.48–1.82)	< 0.001
60–80	4.88 (4.26– 5.58)	3.10 (2.30–4.17)	< 0.001
≥ 80	12.82 (8.95– 18.50)	0.52 (0.06–4.28)	0.55
Absolute platelet count on day 2^b^	1.03 (0.99–1.06)	0.97 (0.94–1.00)	0.07
**Patient with resultant thrombocytopenia**^**c**^			
Relative platelet reduction (%)			
< 20	Reference	Reference	
20–40	1.59 (1.48–1.70)	1.17 (1.10–1.25)	< 0.001
40–60	3.46 (3.16–3.79)	1.69 (1.56–1.83)	< 0.001
60–80	4.78 (4.08–5.59)	2.47 (2.15–2.83)	< 0.001
≥ 80	16.46 (10.10–28.00)	6.55 (4.54–9.46)	< 0.001
Absolute platelet count on day 2^b^	1.59 (1.53–1.65)	1.38 (1.32–1.44)	< 0.001
**Patient with shock**^**d**^			
Relative platelet reduction (%)			
< 20	Reference	Reference	
20–40	1.32 (1.26–1.39)	1.13 (1.07–1.19)	< 0.001
40–60	2.13 (1.98–2.30)	1.59 (1.45–1.73)	< 0.001
60–80	3.30 (2.83–3.83)	2.26 (1.89–2.71)	< 0.001
≥ 80	6.90 (4.83–9.91)	4.82 (3.16–7.34)	< 0.001
Absolute platelet count on day 2 ^b^	1.22 (1.20–1.25)	1.14 (1.12–1.17)	< 0.001
**Patient with respiratory failure**^**e**^			
Relative platelet reduction (%)			
< 20	Reference	Reference	
20–40	1.63 (1.55–1.71)	1.18 (1.12–1.25)	< 0.001
40–60	2.46 (2.26–2.67)	1.36 (1.23–1.50)	< 0.001
60–80	4.86 (4.19–5.64)	2.13 (1.79–2.54)	< 0.001
≥ 80	12.37 (8.36–18.67)	9.21 (5.79–14.66)	< 0.001
Absolute platelet count on day2 ^b^	1.36 (1.33–1.38)	1.24 (1.21–1.27)	< 0.001
**Patient without coagulopathy related complications**			
Relative platelet reduction (%)			
< 20	Reference	Reference	
20–40	1.63 (1.75–1.68)	1.25 (1.21–1.3)	< 0.001
40–60	2.96 (2.8–3.12)	1.85 (1.74–1.97)	< 0.001
60–80	5.89 (5.32–6.53)	3.18 (2.82–3.59)	< 0.001
≥ 80	12.26 (9.36–16.06)	6.13 (4.52–8.31)	< 0.001
Absolute platelet count on day2^b^	1.39 (1.37–1.4)	1.18 (1.17–1.2)	< 0.001

*CI* confidence interval; *SOFA* sequential organ failure assessment.

^a^Variables in the model included patient age, sex, race, Charlson comorbidity index, relative platelet reduction, absolute platelet count on day 2, Acute Physiology and Chronic Health Evaluation (APACHE) IV score, focus of infection, body mass index, nosocomial onset, number of ICU visit, types of unit, region, teaching status, and hospital bed size. Hospital admission as a random intercept to account for clustering by hospital.

^b^Categorized based on Hematology component of SOFA score.

^c^Defined as platelet count ≤ 100 × 10^3^/μL on day 2.

^d^Defined as circulatory component of SOFA score ≥ 3.

^e^Defined as respiratory component of SOFA score ≥ 3.

### Platelet trajectory and mortality

**Figure 3:**
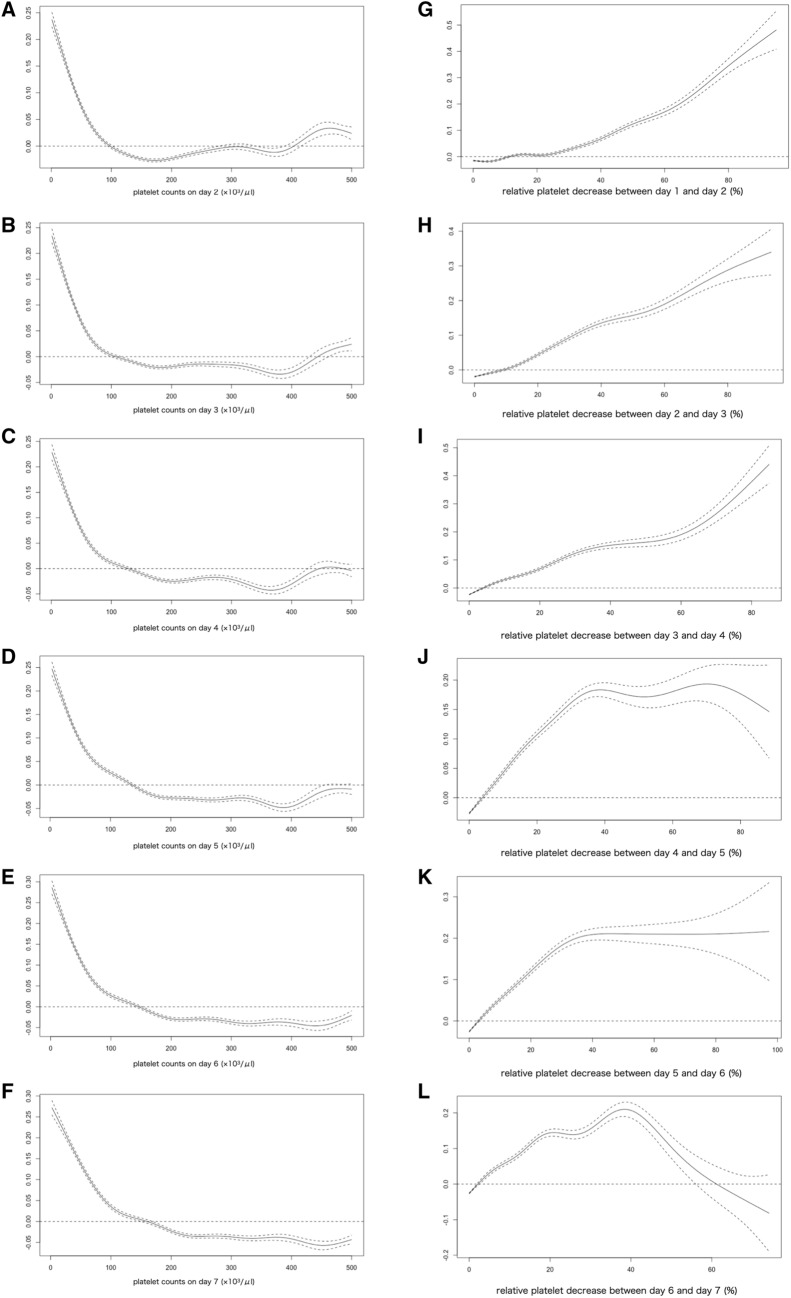
The result of spline regression analysis. (**A**–**F**) the estimated spline function in log odds ratio on the effect of absolute platelet counts on in-hospital mortality. (**G**–**L**) the estimated spline function in log odds ratio on the effect of relative platelet reductions on in-hospital mortality.

In the spline regression analysis using the generalized additive model, the estimated effect of the relative platelet reductions on the in-hospital mortality increased regularly during the initial four days (Fig. 3G–I), while the confidence interval of the estimated effect of a > 40% reduction during days 5–7 widened and became unstable due to the incidence of larger platelet reductions being rare (Fig. 3J–L). The estimated odds on in-hospital mortality increased regularly as the platelet counts decreased to approximately 100,000/μL in any day of the initial seven days (Fig. 3A–F).

### Relative platelet reductions, thrombocytopenia, and coagulopathy-related complications

The results of the multivariate logistic regression analysis for predicting coagulopathy-related complications are shown in Table 3. The optimal cutoff points were determined as platelet reductions of ≥ 11% and absolute platelet counts of ≤ 100,000/μL on day 2 (Fig. S1). While the relative platelet reductions and absolute platelet counts were both associated with coagulopathy related-complications (OR for composite outcome [95% CI], 1.25 [1.19–1.31] and 1.18 [1.07–1.30], respectively), only the relative reductions were associated with the increased odds of thromboembolic events (OR [95% CI]: 1.43 [1.03–1.98]).

**Table 3:** Results of multivariate logistic regression analysis for predicting coagulopathy-related complications.

	Crude Odds ratio (95% CI)	Adjusted odds ratio (95% CI)^a^	*P* value
**For composite outcome**			
Relative platelet decrease ≥ 11%	1.49 (1.42–1.55)	1.25 (1.19–1.31)	< 0.001
Platelet count ≤ 100 × 10^3^/μL on day 2	1.46 (1.39–1.53)	1.16 (1.10–1.22)	< 0.001
**For thrombotic events**			
Relative platelet decrease ≥ 11%	1.42 (1.34–1.50)	1.43 (1.03–1.98)	< 0.001
Platelet count ≤ 100 × 10^3^/μL on day 2	1.05 (0.98–1.12)	0.85 (0.79–0.92)	< 0.001
**For hemorrhagic events**			
Relative platelet decrease ≥ 11%	1.69 (1.58–1.8)	1.31 (1.22–1.40)	< 0.001
Platelet count ≤ 100 × 10^3^/μL on day 2	1.46 (1.39–1.53)	1.58 (1.46–1.70)	< 0.001

Variables in the model included patient age, sex, race, Charlson comorbidity index, relative platelet reduction, absolute platelet count on day2, Acute Physiology and Chronic Health Evaluation (APACHE) IV score, focus of infection, body mass index.

*CI* confidence interval.

### Performance of relative platelet reductions in addition to absolute platelet counts

**Figure 4:**
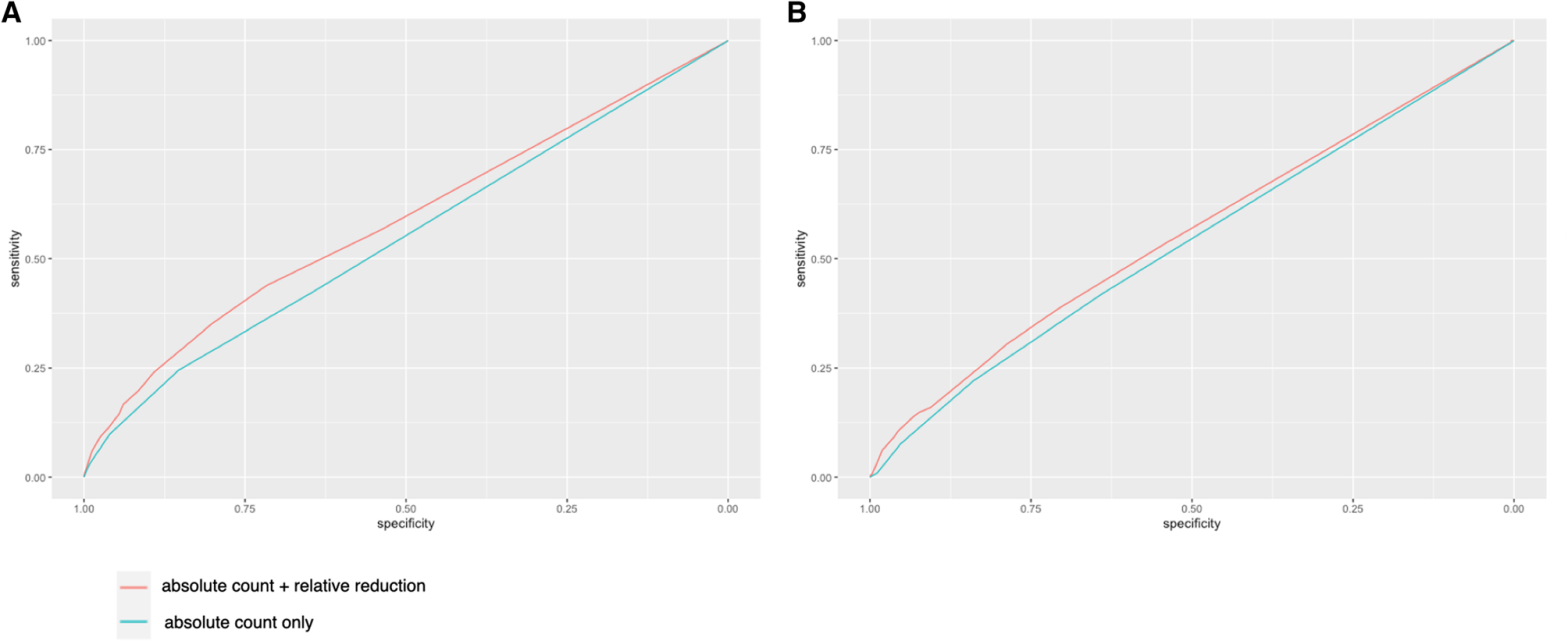
The result of Receiver operating characteristic curve analysis. Predictive validity of platelet counts alone and absolute platelet counts plus relative platelet reductions for in-hospital mortality (**A**) and coagulopathy-related complications (**B**).

The predictive validity for In-hospital mortality using relative platelet reductions in addition to absolute platelet counts showed statistical improvement compared with the use of platelet counts alone (AUROC [95%CI]; 0.598[0.594–0.602] vs. 0.57[0.566–0.577]; *P* < 0.001) (Fig. 4). The predictive validity for coagulopathy-related complications using the relative platelet reductions plus absolute platelet counts was significantly greater than that of platelet counts alone (AUROC [95%CI]; 0.562[0.556–0.569] vs. 0.551[0.542–0.557]; *P* < 0.001).

### Sensitivity analyses

The results of the sensitivity analyses are shown in Table S3 (Additional File 3). The results of the estimated adjusted OR of the platelet reduction rates were similar in the primary analysis, the complete case analysis, the analysis with additional potential risk factors, and the analysis with datasets using alternative inclusion criteria.

## Discussion

To date, this was the largest study to evaluate the prognostic impact of both the magnitude of platelet reductions and the absolute platelet counts in patients with sepsis. The main findings of this study were as follows: (1) the higher the rate at which the platelets were reduced, the greater the estimated mortality rate; this relationship was independent of the absolute platelet count, and (2) platelet reductions were associated with coagulopathy-related complications, which was independent from the resultant thrombocytopenia.

While studies have been conducted thus far on patients who had developed thrombocytopenia during sepsis, limited data were available in terms of the clinical significance of the platelet reduction rates^13,30^. Most of the international coagulopathy criteria for sepsis do not include the rate of platelet reductions as a criterion^12,14,15^. Gando et al. studied 273 patients with sepsis and were the first to report the association between crude mortality and the rate of platelet decrease^13^. Another study evaluated 1,077 critically ill patients and showed that platelet reduction rates ≥ 30% on day four can predict mortality^30^. The results of our study indicated that higher mortality odds were associated with an increasing magnitude of platelet reductions on days 2–7 of sepsis, which was independent of the resultant platelet counts. Furthermore, we identified the association between platelet reductions and the macro thrombotic and bleeding events, independent from thrombocytopenia. On the other hand, the results of the subgroup analysis without coagulopathy-related complications, implicates platelet reductions in the increased mortality odds via immunological and/or micro thrombotic mechanisms outside of the primary hemostatic functions^7–10^. These findings highlight the significance of platelet reduction rates in coagulopathies associated with sepsis.

The severity stratification led to success in showing positive effects of interventions in some previous RCTs in the field of critical care^31,32^. In an experimental study, variations in the phenotypes resulted in unstable RCT conclusions^33^. When designing clinical trials, the platelet reduction rate can be useful in grading the severity of coagulopathies in sepsis and for sample size estimations. Also, absolute platelet counts were not associated with higher odds of thromboembolic events. Targeting optimal patients for the evaluation of anti-coagulation treatment using only the absolute count criterion may be inadequate; the relative platelet reductions may add this value. Furthermore, while using it as a surrogate marker for the development of coagulopathies, preventive interventions for coagulopathies can be assessed. Thus, further studies are required to predict the rapid platelet reductions in the earlier phases of treatment.

The current study used a multicenter, large real-world database in the United States with stratified random sampling^17^. It provided a robust estimation and was not biased by the characteristics and management of sepsis at specific centers. The results can be generalized to patients with sepsis in various ICU settings in high-income countries. However, this study had several limitations. First, excluding earlier deaths resulted in the selection of patients from the less severe group. For those in the early mortality group, it may have been more appropriate to use an earlier timepoint for evaluations, such as within the first 3–6 h. Second, the real-world data did not include further details on coagulation markers (e.g., D-dimers, anti-thrombin activities, and the presence of plasminogen activator inhibitor-1), which did not allow for further evaluations of the usefulness of platelet reductions in comparison with these markers. Third, the severity of the complications was not reported. Prospective studies that evaluate the incidences and severities of coagulopathic complications in high-risk populations (i.e. those patients with rapid platelet reductions) are warranted.

## Conclusion

Our findings suggest that the identification of the magnitude of relative platelet reductions was a better tool for the stratification of the risk of mortality and coagulopathy-related complications in combination with resultant absolute platelet counts. Thus, it may be a more plausible criterion for the assessment of coagulopathies in sepsis.

## Supplementary Information

Supplementary Table S1.

Supplementary Table S2.

Supplementary Table S3.

Supplementary Figure S1.

Supplementary Legends.

## Data Availability

The data used for this manuscript are available from the eICU Collaborative Research database: https://eicu-crd.mit.edu/.

## Abbreviations

ICUs: Intensive care units
DIC: Disseminated intravascular coagulation
SOFA: Sequential Organ Failure Assessment
RECORD: REporting of studies Conducted using Observational Routinely collected health Data
APACHE: Acute Physiology and Chronic Health Evaluation
RCTs: Randomized control trials
