# Supplementary material for: Relative platelet reductions provide better pathophysiologic signatures of coagulopathies in sepsis
Source: Sci Rep. 2021 Jul 7;11:14033. doi: 10.1038/s41598-021-93635-5 (PMC8263719; doi:10.1038/s41598-021-93635-5)
Supplement: Supplementary file 1 — Supplementary Table S1. [file 41598_2021_93635_MOESM1_ESM.docx]

**Table S1.** Definition and location of extracted diagnoses from the database

| **Definition** | **Table** | **Column** | **Data** |
| --- | --- | --- | --- |
| Thrombosis | Diagnosis | Diagnosis string | “intracranial venous thrombosis”, “pulmonary embolism”, “vascular thrombosis”, “peripheral vascular ischemia”, “arterial thromboembolism”, “thrombectomy”, “embolectomy”, “massive pulmonary embolus”, “ischemic enterocolitis”, “myocardial infarction”,” mesenteric ischemia” |
| Hemorrhage | Diagnosis | Diagnosis string | “subarachnoid hemorrhage”, “Intracerebral hemorrhage”, “intraventricular hemorrhage”, “abdominal compartment syndrome\|hemorrhage related”, “hemorrhage of liver”, “post craniotomy\|for hemorrhage”, “pulmonary hemorrhage”, “wound hemorrhage”, “hemorrhage – postpartum”, “vaginal hemorrhage”, “internal hemorrhage”, “acute blood loss anemia”, “GI bleeding”, “hemorrhagic stroke”, “bleeding and red blood cell disorders\|hemorrhage”, “cardiac surgery\|hemorrhage”, “thoracic surgery\|hemorrhage”, “vascular surgery\|hemorrhage”, “general surgery postop issues\|hemorrhage”, “hypovolemic shock\|hemorrhagic” |
