# Supplementary material for: Relative platelet reductions provide better pathophysiologic signatures of coagulopathies in sepsis
Source: Sci Rep. 2021 Jul 7;11:14033. doi: 10.1038/s41598-021-93635-5 (PMC8263719; doi:10.1038/s41598-021-93635-5)
Supplement: Supplementary file 2 — Supplementary Table S2. [file 41598_2021_93635_MOESM2_ESM.docx]

**Table S2.** Baseline characteristics and number of patients with missing data

| **Factor** | **Overall** |
| --- | --- |
| **Total, n** | 26176 |
| **Age, years, mean (SD)**  missing data, n (%) | 66.16 (15.4)  1 (0.00) |
| **Admission height, cm, mean (SD)**  missing data, n (%) | 168.6 (11.8)  187 (0.01) |
| **Admission weight, kg, mean (SD)**  missing data, n (%) | 84.07 (29.5)  216 (0.01) |
| **Male, n (%)** | 13514 (51.6) |
| **Ethnicity, n (%)** |  |
| Caucasian | 20370 (77.8) |
| African American | 2752 (10.5) |
| Asian | 418 (1.6) |
| Hispanic | 1057 (4.1) |
| Native American | 216 (0.8) |
| Other/unknown | 1363 (5.2) |
| **First ICU visit, n (%)** | 22267 (85.1) |
| **Focus of infection, n (%)** |  |
| Abdominal | 3596 (13.7) |
| Pulmonary | 13611 (52.0) |
| Soft tissue | 1751 (6.7) |
| Urinary tract | 3907 (15.0) |
| Others/unknown | 3291 (12.6) |
| **Charlson comorbidity index, median (IQR)** | 4 (2-6) |
| **Comorbidities, n (%)** |  |
| Chronic heart failure | 5056 (19.3) |
| Myocardial infarction | 2250 (8.6) |
| Chronic obstructive pulmonary disease | 6834 (26.1) |
| Stroke | 2664 (11.8) |
| Chronic renal failure | 538 (2.1) |
| Mild liver disease | 680 (2.6) |
| Moderate-severe liver disease | 258 (1.0) |
| Dementia | 1528 (5.9) |
| Diabetes mellites with complication | 3808 (14.54) |
| Peripheral vascular disease | 1379 (5.3) |
| Peptic ulcer disease | 797 (3.1) |
| Collagen vascular disease | 803 (3.1) |
| Hypothyroid disease | 2779 (10.6) |
| Metastatic cancer | 837 (3.2) |
| Hematologic malignancy | 789 (3.0) |
| Acquired immune deficiency syndrome | 75 (0.32) |
| **Unit type, n (%)** |  |
| Medical ICU | 3513 (13.4) |
| Medical-Surgical ICU | 17004 (65.0) |
| Surgical ICU | 1266 (4.8) |
| Cardiac ICU | 1604 (6.1) |
| CCU-Cardio-Thoracic ICU | 1458 (5.6) |
| Cardiac-Thoracic ICU | 233 (0.9) |
| Cardiac Surgical ICU | 483 (1.8) |
| Neurological ICU | 615 (2.4) |
| **Facility bed capacity, n (%)** |  |
| 0-100 | 1535 (5.42) |
| 100-249 | 5928 (22.7) |
| 250-499 | 6680 (27.1) |
| ≧500 | 9067 (33.8) |
| missing data | 2966 (11.1) |
| **Platelet count on day 1, ×10^3^/μL, median (IQR)** | 181 (125–251) |
| **Platelet count on day 2 ×10^3^/μL, median (IQR)**  missing data, n (%) | 174 (117–242)  4122 (15.7) |
| **Rate of platelet decrease, %, median (IQR)** | 4.6 (–7.1– 17) |
| **SOFA score, median (IQR), median (IQR)** | 7 (5–10) |
| SOFA score without hematology component | 4 (3–7) |
| Circulatory component | 0 (0–1) |
| Hematology component | 0 (0–1) |
| Hepatology component, median (IQR)  missing data, n (%) | 0 (0–1)  9767 (38.1) |
| Neurological component  missing data, n (%) | 1 (0–3)  5408 (20.7) |
| Renal component  missing data, n (%) | 1 (0–3)  993 (3.79) |
| Respiratory component  missing data, n (%) | 2 (2–3)  9560 (38.1) |
| **APACHE IV score, median (IQR)**  missing data, n (%) | 64 (50–81)  3816 (14.6) |
| **APS III score, median (IQR)**  missing data, n (%) | 50 (38–66)  3820 (14.6) |
| **Treatment on day 1**, **n (%)** |  |
| Mechanical ventilation | 1925 (7.3) |
| Heparin | 7808 (29.8) |
| Cefepime | 1403 (5.4) |
| Ceftriaxone | 1805 (6.9) |
| Levofloxacin | 3366 (12.9) |
| Meropenem | 705 (2.7) |
| Metronidazole | 1141 (4.4) |
| Piperacillin/tazobactam | 5660 (21.6) |
| Vancomycin | 9214 (35.2) |
| Renal replacement therapy | 1108 (4.2) |
| **Coagulopathic complications, n (%)** |  |
| Thrombosis | 139 (0.5) |
| Hemorrhage | 145 (0.6) |
| **Length of hospital stay, days, median (IQR)** | 7.4 (4.7–12.1) |
| **In-hospital mortality, n (%)** | 3208 (12.3) |

SD, standard deviation; IQR, interquartile range; SOFA, sequential organ failure assessment; APACHE, acute physiology and chronic health evaluation; APS, acute physiology score.
