# Supplementary material for: Relative platelet reductions provide better pathophysiologic signatures of coagulopathies in sepsis
Source: Sci Rep. 2021 Jul 7;11:14033. doi: 10.1038/s41598-021-93635-5 (PMC8263719; doi:10.1038/s41598-021-93635-5)
Supplement: Supplementary file 3 — Supplementary Table S3. [file 41598_2021_93635_MOESM3_ESM.docx]

**Table S3.** The sensitivity analyses results

|  | Complete case analysis (n = 19075) | | Model with additional potential risk factors ^a^ (n=26176) | | Explicit criteria cohort (n = 17760) | |
| --- | --- | --- | --- | --- | --- | --- |
|  | OR (95% CI) | *P* value | OR (95% CI) | *P* value | OR (95% CI) | *P* value |
| Relative platelet reduction (%) |  |  |  |  |  |  |
| <20 | Reference |  | Reference |  | Reference |  |
| 20–40 | 1.27 (1.12–1.44) | <.001 | 1.26 (1.22–1.31) | <.001 | 1.27 (1.22–1.33) | <.001 |
| 40–60 | 1.90 (1.53–2.35) | <.001 | 1.84 (1.73–1.95) | <.001 | 1.83 (1.72–1.96) | <.001 |
| 60–80 | 3.41 (2.24–5.20) | <.001 | 2.94 (2.62–3.30) | <.001 | 2.98 (2.63– 3.38) | <.001 |
| ≥80 | 8.31 (2.75–25.08) | <.001 | 5.83 (4.24–8.01) | <.001 | 6.15 (4.36–8.68) | <.001 |
| Absolute platelet count on day 2 ^b^ | 1.20 (1.15–1.26) | <.001 | 1.19 (1.18–1.21) | <.001 | 1.17 (1.16–1.19) | <.001 |

OR, odds ratio; CI, confidence interval.

^a^ Additional covariates include antibiotics administered on day 1 of hospital admission (cefepime, ceftriaxone, levofloxacin, meropenem, metronidazole, piperacillin/tazobactam, vancomycin), heparin and renal replacement therapy.

^b^ Categorized based on the hematology component of the Sequential Organ Failure Assessment score
