# Supplementary figures and images for: Relative platelet reductions provide better pathophysiologic signatures of coagulopathies in sepsis
Source: Sci Rep. 2021 Jul 7;11:14033. doi: 10.1038/s41598-021-93635-5 (PMC8263719; doi:10.1038/s41598-021-93635-5)

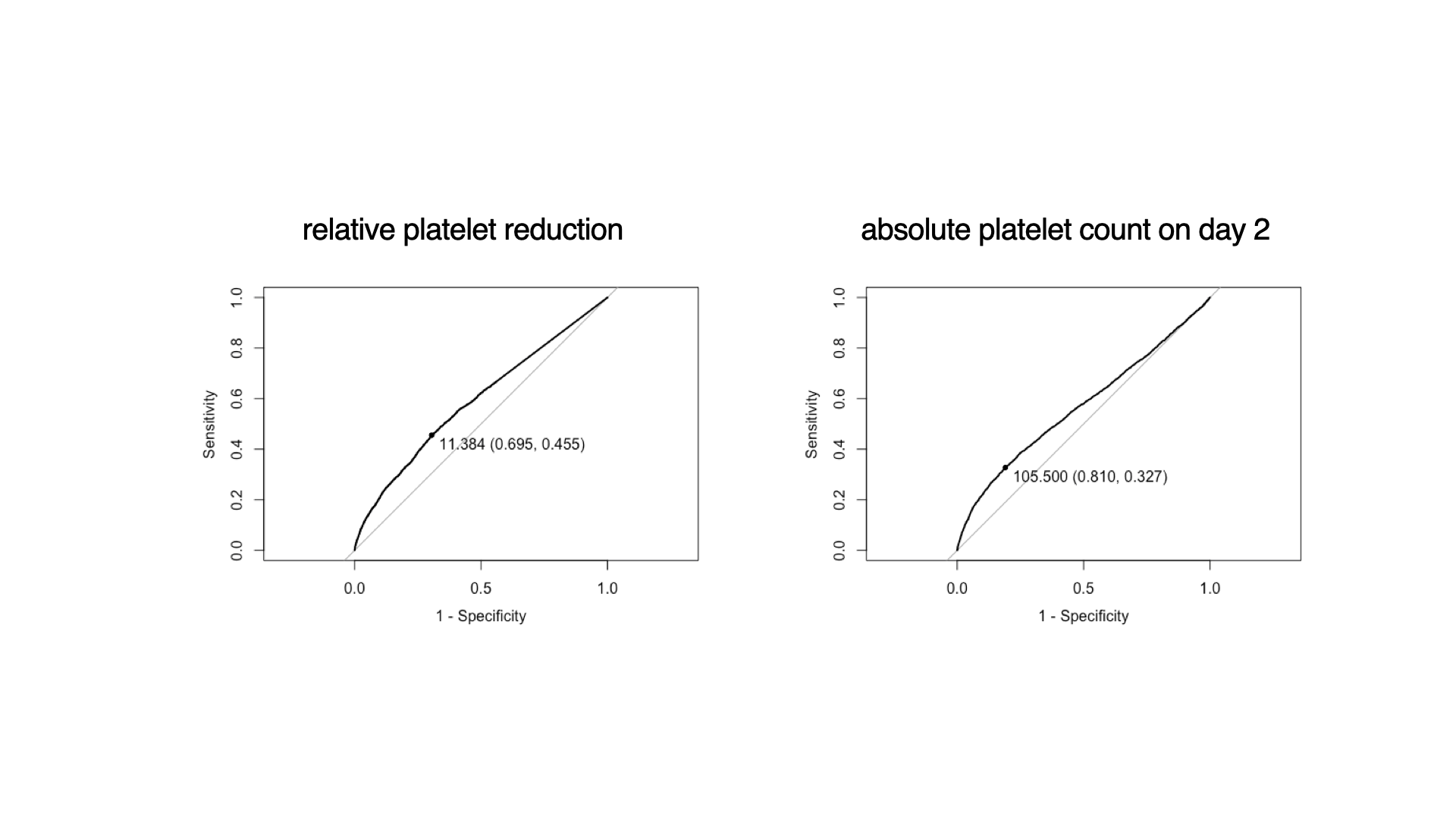

Supplement: Supplementary file 4 — Supplementary Figure S1. [file 41598_2021_93635_MOESM4_ESM.tiff]
