# Supplementary material for: Relative platelet reductions provide better pathophysiologic signatures of coagulopathies in sepsis
Source: Sci Rep. 2021 Jul 7;11:14033. doi: 10.1038/s41598-021-93635-5 (PMC8263719; doi:10.1038/s41598-021-93635-5)
Supplement: Supplementary file 5 — Supplementary Legends. [file 41598_2021_93635_MOESM5_ESM.docx]

**Supplementary information**

**Additional file 1: TableS1.** Definition and location of extracted diagnoses from the database

**Additional file 2: TableS2.** Baseline characteristics and number of patients with missing data

**Additional file 3: TableS3.** Result of the sensitivity analyses

**Additional file 4: FigureS1.** The cutoff determination using the ROC curve analysis and Youden index
